# Regulation of Intestinal Immune Response by Selective Removal of the Anterior, Posterior, or Entire Pituitary Gland in *Trichinella spiralis* Infected Golden Hamsters

**DOI:** 10.1371/journal.pone.0059486

**Published:** 2013-03-15

**Authors:** Rosalía Hernández-Cervantes, Andrés Quintanar-Stephano, Norma Moreno-Méndoza, Lorena López-Griego, Valeria López-Salazar, Romel Hernández-Bello, Julio César Carrero, Jorge Morales-Montor

**Affiliations:** 1 Departamento de Inmunología, Instituto de Investigaciones Biomédicas, Universidad Nacional Autónoma de México, México Distrito Federal, México; 2 Departamento de Fisiología y Farmacología, Centro de Ciencias Básicas, Universidad Autónoma de Aguascalientes, Aguascalientes, Aguascalientes, México; 3 Departamento de Biología Celular, Instituto de Investigaciones Biomédicas, Universidad Nacional Autónoma de México, México Distrito Federal, México; 4 Departamento de Microbiología, Facultad de Medicina. Universidad Autónoma de Nuevo León. Monterrey, Nuevo León, México; Virginia Tech, United States of America

## Abstract

The influence of anterior pituitary hormones on the gastrointestinal tract of humans and animals has been previously reported. Hypophysectomy (HYPOX) in the rat causes atrophy of the intestinal mucosa, and reduction of gastric secretion and intestinal absorption, as well as increased susceptibility to bacterial and viral infections. However, to our knowledge, no findings have been published concerning the immune response following HYPOX during worm infection, particularly that which is caused by the nematode *Trichinella spiralis*. The aim of this work was to analyze the effects of total or partial HYPOX on colonization of *T. spiralis* in the intestinal lumen, together with duodenal and splenic cytokine expression. Our results indicate that 5 days post infection, only neurointermediate pituitary lobectomy (NIL) reduces the number of intestinally recovered *T. spiralis* larvae. Using semiquantitative inmunofluorescent laser confocal microscopy, we observed that the mean intensity of all tested Th1 cytokines was markedly diminished, even in the duodenum of infected controls. In contrast, a high level of expression of these cytokines was noted in the NIL infected hamsters. Likewise, a significant decrease in the fluorescence intensity of Th2 cytokines (with the exception of IL-4) was apparent in the duodenum of control and sham infected hamsters, compared to animals with NIL surgeries, which showed an increase in the expression of IL-5 and IL-13. Histology of duodenal mucosa from NIL hamsters showed an exacerbated inflammatory infiltrate located along the lamina propria, which was related to the presence of the parasite. We conclude that hormones from each pituitary lobe affect the gastrointestinal immune responses to *T. spiralis* through various mechanisms.

## Introduction

In addition to the hypothalamic control of adenohypophysial and neurohypophysial hormones (neuroendocrine system), which regulate body growth, cell differentiation, maturation, metabolism, reproduction, stress, aging, lactation, and hydro electrolytic balance, a great deal of information indicates that in the maintenance of body homeostasis, pituitary hormones interact with the immune system, as well as during bacterial, parasitic, viral, and auto-antigenic challenges [1]–[7] to overcome infectious and inflammatory diseases. The mediators in the immunoneuroendocrine interactions, include cytokines from immune and inflammatory cells, receptors, hormones, neuropeptides and neurotransmitters. Communication between the immune and neuroendocrine system begins when cytokines released during inflammation interact with cytokine receptors in peripheral nerves, or when cytokines from the inflammatory loci are blood-borne to the several nerve nuclei in the central nervous system (CNS), where they interact with cytokine receptors. After neural integration (mainly in the hypothalamus), the autonomous nervous system and the hypophysiotropic hormones regulate pituitary hormone secretion, which together with local cytokines integrate an adequate immune response. Thus, after the activation of the hypothalamic-pituitary-adrenocortical (HPA) axis through glucocorticoids (cortisol and corticosterone), HPA axis has a modulator/suppressor effect on the immune system preventing an excessive immune response [8], [9]. The HPA axis is also involved in the adaptation and maintenance of homeostasis during critical illness, and during bacterial, parasitic, viral, and autoimmune/inflammatory diseases [10]–[13]. Growth hormone (GH), prolactin (PRL), and the neurohypophysial arginine vasopressin (AVP) are immunomodulators/immunostimulators [4], [14]–[21], whereas sex hormones of the hypothalamic-pituitary-gonadal (HPG) axis affect the susceptibility of the immune system when facing immune challenges, thus determining the development of infections and autoimmune diseases [22].

Most information regarding the neuroimmunoendocrine interactions is related to systemic immune responses [2], [4], [5], [15], [23], whereas less is known about the role of the neuroendocrine and intestinal mucosa-gut associated lymphoid tissue (GALT) systems [24]–[27]. In intact rats and mice, GH, IGF-1 and PRL enhance resistance to *Salmonella serovar typhimurium* infections by increasing phagocytosis and intracellular destruction of bacteria by macrophages. It was also found that GH and PRL stimulate chemotaxis of peritoneal granulocytes [27]–[31]. These findings are similar in hypophysectomized (HYPOX) rats orally infected with sub-lethal doses of *Salmonella serovar typhimurium*, which developed an increased colonization of Peyer patches and spleen (Campos-Rodríguez et al, 2006), whereas the intraperitoneal infection was reversed to normal when GH was administrated [30]. In rats subjected to HYPOX, neuro-intermediate pituitary lobectomy (NIL), and anterior pituitary lobectomy (AL), AVP plays an important role as immunomodulatory hormone on innate and acquired immunity [1], [3], [21], [32]–[34]. Regarding the ileal mucosa-GALT, evidence indicates that in basal conditions, AVP regulates the number of CD4 and CD8 lamina propria lymphocytes, IgM and IgA producing cells, and IgA secretion, as well as intraepithelial CD8+ T cells numbers [1], [3]. Whereas, in response to sub-lethal doses of *Salmonella serovar typhimurium* [1], NIL animals, with low AVP serum levels, developed a higher *Salmonella serovar typhimurium* colonization in the Peyer patches and spleen, and decreased intestinal IgA and serum anti salmonella IgM and IgG levels [1].

Nematodes from the *Trichinella* genus, such as *Trichinella spiralis,* the causal agent of porcine and human trichinellosis, are highly evolved parasites which have developed diverse mechanisms for survival within the host that facilitate colonization. *Trichinella spiralis* is an intracellular parasitic nematode which invades mammalian striated muscles and is responsible for trichinellosis, a zoonosis caused by consumption of raw or undercooked meat from infected animals (e.g. pork). It has been estimated that way more than 11 million people worldwide are likely to be infected [35]. The *T. spiralis* life-cycle is completed within a single host and the parasite resides in two distinct intracellular habitats. The infective stage of *T. spiralis* is part of a nurse cell-larva complex found in the striated muscle of prey eaten by carnivores, whereas the female adult form is embedded into epithelial cells of the intestinal mucosa. It is known that endocrine factors, related to gender and age of the host, play an important role in determining the nature of the immune response [36], [37], or directly affecting the parasite. Thus, hormones play key roles in determining susceptibility to trichinellosis in two ways: by providing a protective immune response, or by having a direct effect on parasite development [38], [39]. Males are generally more susceptible than females to *T. spiralis* infections [40]. However, it is unknown whether this effect is mediated by an immunoendocrine interaction. Recent experimental evidence suggests that steroids and protein hormones may exert their effects directly upon the parasite [38], which may be able to exploit the host’s hormonal microenvironment for its exclusive benefit [41], [42]. In fact, the hormonal microenvironment in an immunocompetent host is so important, that an inadequate hormonal environment may lead to apoptosis in parasite cells, as has been proposed [43]. The hormonal microenvironment also modulates gene expression of female and male *S. mansoni* adult parasites [44].

To our knowledge, no data have been published indicating whether hypophysial hormones are able to affect intestinal immune responses to gastrointestinal worm infection. The aim of our work is to analyze the effects of adenohypophysial or neurohypophysial pituitary hormones deficiency, so we studied the effects of adenohypophysial, neurointermediate, and total hypophysectomy on duodenal *T. spiralis* colonization, intestinal histological changes, intestinal cytokine localization, and splenic cytokines expression.

## Materials and Methods

### Ethics Statement

Animal care and experimentation practices at the Instituto de Investigaciones Biomédicas and the Universidad Autónoma de Aguascalientes are constantly evaluated by the Institutés Animal Care and Use Committee, adhering to the official Mexican regulations (NOM-062-ZOO-1999). Mexican regulations are in strict accordance with the recommendations in the Guide for the Care and Use of Laboratory Animals of the National Institute of Health (NIH and The Weatherall Report) of the USA, to ensure compliance with established international regulations and guidelines. The protocol was approved by the Committee on Ethics of Animal Experiments of the Instituto de Investigaciones Biomédicas (Permit Number: 2009–16). The hamsters were sacrificed using sodium pentobarbital anesthesia to obtain parasites and tissues. Efforts were made to minimize suffering.

### Animals

Sixty three month old male Syrian golden hamsters (*Mesocricetus auratus*) from our colony were used. The animal room was at a controlled temperature (22–24°C) and 12 hours light-dark conditions (lights on at 0700 hrs). The diet consisted of Purina rat chow and tap water *ad libitum*. In the anterior pituitary lobectomized (AL) and hypophysectomized (HYPOX) hamsters, diet was complemented with flavored cookies, sliced apples, and 5% sugar in drinking water.

### Groups

Animals were divided into the following groups of 10 animals each (5 animals per cage): 1) Non-operated, non-infected (Intact), 2) Intact-non operated-Infected (Infected), 3) Sham operated-infected (SHAM), 4) Anterior pituitary lobectomized-infected (AL), 5) Neurointermediate pituitary lobectomized-infected (NIL) and 6) Total hypophysectomized-infected (HYPOX). Animals were weighed before surgeries and subsequently once a week until sacrifice (29 days after surgery).

### Surgeries

Animals were anesthetized with methyl ether and the trachea was cannulated per os. In order to prevent excessive secretion in the respiratory tract, 0.06-mg atropine was administered subcutaneously 15 minutes prior to anesthesia. SHAM, NIL, AL, and HYPOX surgeries were performed under a stereoscopic dissecting microscope, through the parapharyngeal approach. A hole was drilled in the ventral aspect of the occipital-sphenoid joint until the pituitary capsule was predominantly exposed. In SHAM animals, the operation was terminated when the pituitary capsule was surgically opened and the pituitary gland became directly visible. In the case of NIL, in order to observe the neural lobe, the posterior border of the pituitary capsule was cut and the caudal border of the gland lifted; after a direct view of the neural lobe it was carefully aspirated with a special curved needle. In the case of AL, the pituitary capsule was widely opened, and the adenohypophysial lobe cut into two halves; each half was then carefully aspirated, thus leaving the posterior lobe, the infundibular stalk, and the hypothalamic median eminence untouched. In the case of HYPOX, the entire gland was removed. The total time of anesthesia did not exceed 15 min and full recovery occurred within 30–60 min. Following surgery, all operated animals were injected with penicillin (Penprocilina; 5000 IU intramuscularly; Lakeside. México) once a day for three days. The methods employed for HYPOX have been previously described by Alvarez-Buylla in 1991 [45], and for NIL by Ben-Jonathan and Peters in 1982 [46], and Mena et al, in 1996 [47].

### Experimental Infections

*Trichinella spiralis* (ISS 406) has been maintained in our laboratory by serial passage infections in BALB/c mice. The infective-stage muscular larvae (ML) were recovered from experimentally infected mice at 30 days post infection. Muscles were digested by a standard pepsin-hydrochloric acid digestion method. Larvae were recovered and counted under a stereoscopic microscope.

Three weeks after surgeries, all animals with the exception of the intact group were infected with 1500 ML by intragastric route. Five days post infection, the adult worms were recovered from the small intestine. Hamsters were euthanized using (2,2,2-trifluoro-1-[trifluoromethyl] ethyl fluoromethyl ether, (Sevorane)). The small intestine was dissected, cut longitudinally, and washed twice in PBS 1X. The intestine was then cut into small pieces and incubated in sterile PBS 1X for 3 hrs at 37°C. Following incubation, parasites were collected and counted under a stereoscopic microscope.

### TNFα, IL-10, and IL-6 Cytokine Expression in the Spleen

Splenic tissue samples were placed on Trizol reagent (Invitrogen, Carlsbad, California). Total RNA extraction was as follows: the spleen was disrupted in Trizol reagent (1 ml/0.1 g tissue), and 0.2 ml of chloroform was added per ml of Trizol. The aqueous phase was recovered after 10 min of centrifuge at 13000 rpm. RNA was precipitated with isopropyl alcohol, washed with 75% ethanol, and dissolved in RNAse-free water. RNA concentration was determined by absorbance at 260 nm, and its integrity was verified following electrophoresis on 1.0% denaturing agarose gel in the presence of 2.2 M formaldehyde. Total RNA samples were immediately reverse-transcribed, using M-MLV Retrotranscriptase system and dT_12–18_ primer (Invitrogen, USA). Then, cDNA was specifically amplified by semi-quantitative PCR, using TaqDNA polymerase (Biotecnologías Universitarias, UNAM. México) and hamster-specific primers to detect TNFα, IL-6, and IL-10. Briefly, the 50 µl of PCR reaction volume included 10 µl of previously synthesized cDNA, 5 µl of 10X PCR-buffer (Perkin-Elmer, USA), 1 mM of MgCl, 0.2 mM of each dNTP, 0.05 µM of each primer, and 2.5 units of TaqDNA polymerase (Biotecnologias Universitarias, UNAM. Mexico). Following an initial denaturation step at 95°C for 5 min, temperature cycling was as follows: 95°C for 30 seconds, between 51°C and 62°C (depending on primer sequence) for 45 seconds and 72°C for 45 seconds during 35 cycles. An extra extension step was completed at 72°C/10 min for each gene. The 50 µl from the PCR reaction were electrophoresed on 2% agarose gel, stained with ethidium bromide in the presence of a 100 bp ladder as a molecular weight marker (Gibco, BRL, NY). 18S-ribosomal RNA was used as a gene to control constitutive expression. Relative expression rate of each amplified gene was obtained by optical density analysis (OD), using the 18S-ribosomal RNA as a constitutive control.

### Histological Examination

Non incubated duodenum samples from each animal were fixed in 4% paraformaldehyde (J.T. Baker, México), dehydrated, and embedded in paraffin. Cross-sections of 6 µm were cut and mounted on poly-L-lysine (Sigma, St Louis, MO, USA) coated slides for histological and immunohistochemical evaluation. The histologic findings were evaluated on hematoxylin-eosin (H-E) stained sections and immunofluorescence stained sections (*vide infra* for details) under light microscope at 6×, 10×, 40×, and 100× magnification.

### Duodenum Cytokine Localization by Immunohistochemistry Fluorescence

Antibodies anti-mouse IL-2, IL-1β and IL-5 produced in rabbit, anti-mouse INFγ, TNFα, IL-12, IL-10, IL-6 and IL-13 produced in goat, and anti-mouse IL-4 produced in rat were used as primary antibodies (Santa Cruz, Biotechnology, USA). Anti-rabbit IgG, anti-goat IgG, and anti-rat conjugated with rhodamine (TRITC) (ZIMED Laboratories Inc., USA) were used as secondary antibodies. Duodenal slides were processed for immunohistochemistry. Briefly, rehydrated sections were treated with 1% Triton X-100, after blocking with 1% albumin (BSA), and then incubated with the primary antibody overnight, at 4°C diluted in 1∶200 BSA. After rinsing in PBS, sections were incubated with the secondary antibody for 1 h at room temperature, diluted in 1∶100 BSA, washed in PBS, and embedded in DAKO fluorescent mounting medium (DAKO, USA). Slides processed without the primary antibody were used as negative controls. For each group, several field images of duodenum were captured and analyzed by semiquantitative immunofluorescence laser confocal microscopy. In brief, TIFF images were acquired with the TCS-SP1 software and imported into Image Pro Plus (Media Cybernetics, Inc. USA), in order to subsequently evaluate the immunofluorescence intensity. For each image, representative areas were selected and the exposure times kept constant, so that the intensity of fluorescence could be quantified and expressed as the mean pixel intensity for that region. At least six randomly selected areas were analyzed from each animal.

### Experimental Design and Statistical Analysis

Results were determined in 2 independent experiments (five animals each). Dependent variables included the number of parasites, the proliferation index, IL-2, IL-1β, IL-5, INFγ, TNFα, IL-12, IL-10, IL-6, and IL-13 expression, as well as the number of inflammatory foci. The independent variable was surgery. Data from 2 replicates (n = 5) of each experimental group were expressed as an average +/− standard deviation, and analyzed by means of one way-ANOVA, and Tukey as post hoc test. Differences were considered significant when p<0.05.

## Results

### Body Weights

As compared with the Intact group, no significant changes were observed in Infected, SHAM, or NIL groups, whereas a gradual but significant decrease in body weight was apparent in AL and HYPOX groups. Thus, at the end of experiment these groups lost 20% of body weight as compared with the remaining groups (not shown).

### Intestinal Adult Parasite Recovery

**Figure 1:**
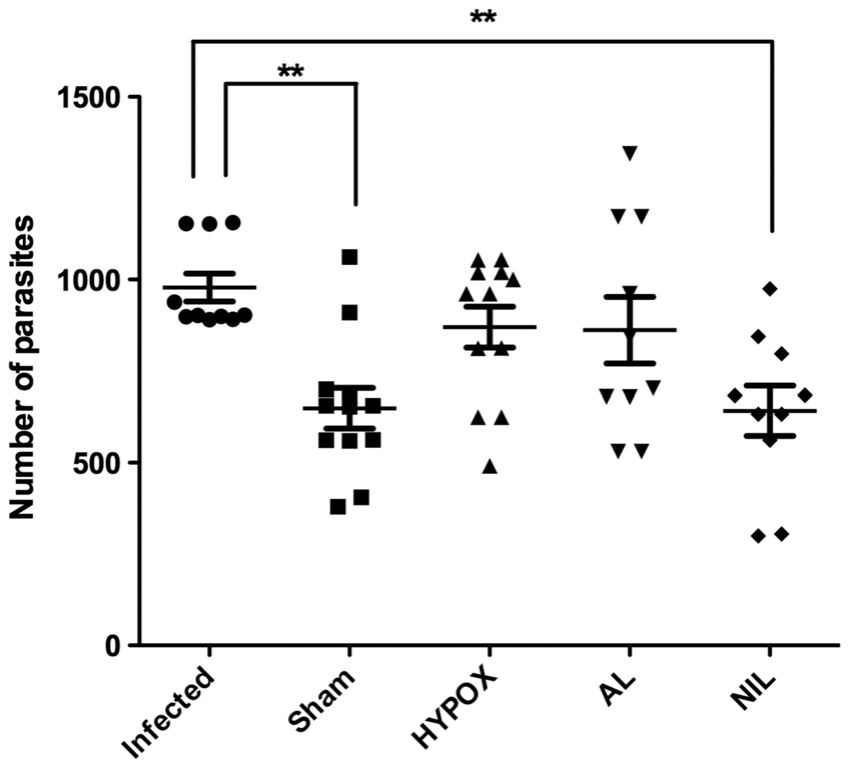
Number of intestinal parasites recovered from the small intestine of hamsters infected with *Trichinella spiralis*. Hamsters were infected with 1500 ML of *T. spiralis* and sacrificed at 5 days pi. The small intestine was obtained, and then longitudinally cut into small pieces for posterior incubation at 37°C for 3 hours to recover larvae. One-way ANOVA. N = 10−12. ** = P<0.05.

As compared with the infected animals, in all operated groups, a decreased number of parasites were recovered from the intestine, although significant differences were apparent only in SHAM and NIL groups (p<0.01) (Fig. 1). No significant differences in parasite numbers were apparent between SHAM, HYPOX, AL, and NIL groups (Fig. 1).

### Spleen Weights

**Figure 2:**
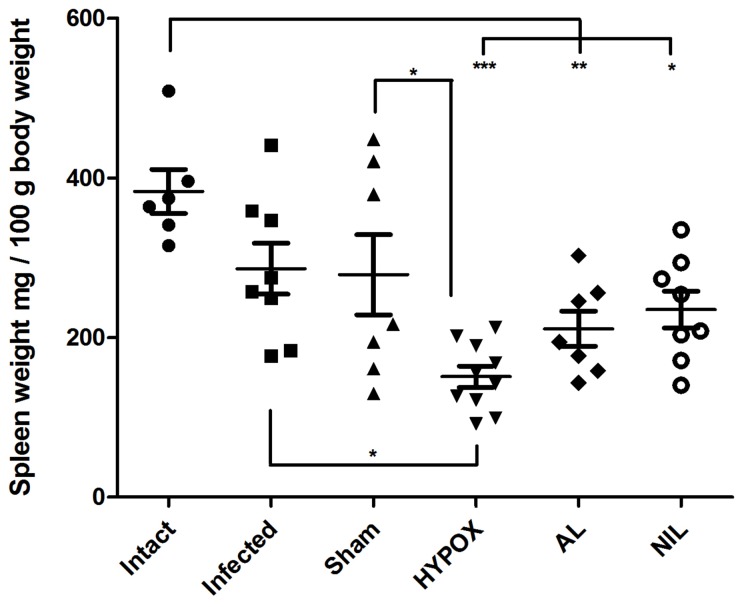
Effects of neurosurgery on spleen characteristics. (A) Only HYPOX animals showed a tendency to decrease in spleen weight, and no significant differences in spleen weight were observed in any other group. One-way ANOVA. N = 6−10. *** = P<0.001, ** = P<0.01, * = P<0.05.

To assess the relationship between the several hormonal milieus with the immune response to *T. spiralis* intestinal infection, spleens from all groups were weighed. Figure 2 shows that, as compared with the Intact animals, all surgeries induced a decreased spleen weight, although significant differences were appreciated only in HYPOX, AL, and NIL animals (p<0.001, 0.01 and 0.05 respectively).

### Spleen Cytokines mRNA Expression

**Figure 3:**
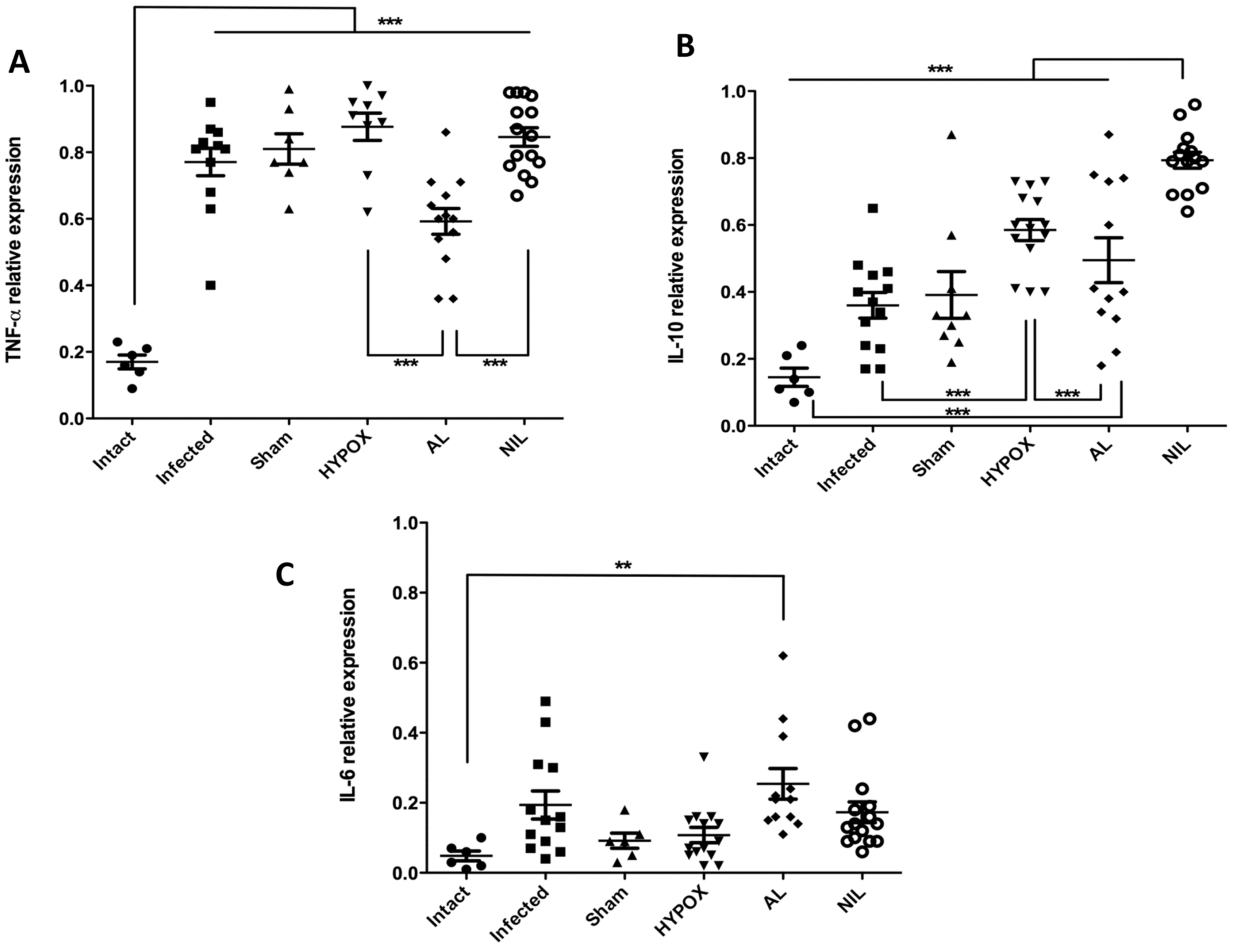
Expression of TNFα, IL-10 and IL-6 in spleen of hamsters experimentally infected with *T. spiralis* at 5 days pi. The 18S was used as control gene of constitutive expression. Optical densitometry (OD) was obtained as a relationship between the genes of interest and 18S, and reported as mean +/− standard deviation. One-way ANOVA. N = 6−15. *** = P<0.001, **P<0.05.

To assess the effects of the several hormonal milieus on systemic immunological mechanism involved in the intestinal immune response to *T. spiralis*, TNFα, INFγ, IL-10, IL-6, and IL-12 spleen cytokines were assessed by RT-PCR. Of these, only TNFα, IL-10, and IL-6 cytokines were expressed. As compared with the Intact group, TNFα expression was significantly increased in all infected groups (p<0.001) (Fig. 3A), whereas AL animals expressed significantly less TNFα than the HYPOX and NIL groups (p<0.001). Figure 3B shows that, in comparison with the Intact group, IL-10 expression was increased in all infected groups. However, the different expression of this cytokine was pituitary hormonal milieu-dependent. Thus, whereas no significant differences were apparent between Infected, SHAM and AL groups, NIL animals developed a significant higher expression of IL-10 than the remaining groups. As compared with the Intact, Infected, and AL groups, HYPOX animals expressed a significant increase in IL-10. Figure 3C shows that, except the mild but significant increase of IL-6 in AL animals, no significant expression of this cytokine occurred in the remaining groups.

### Intestinal Cytokines Assessed by Immunofluorescence

**Figure 4:**
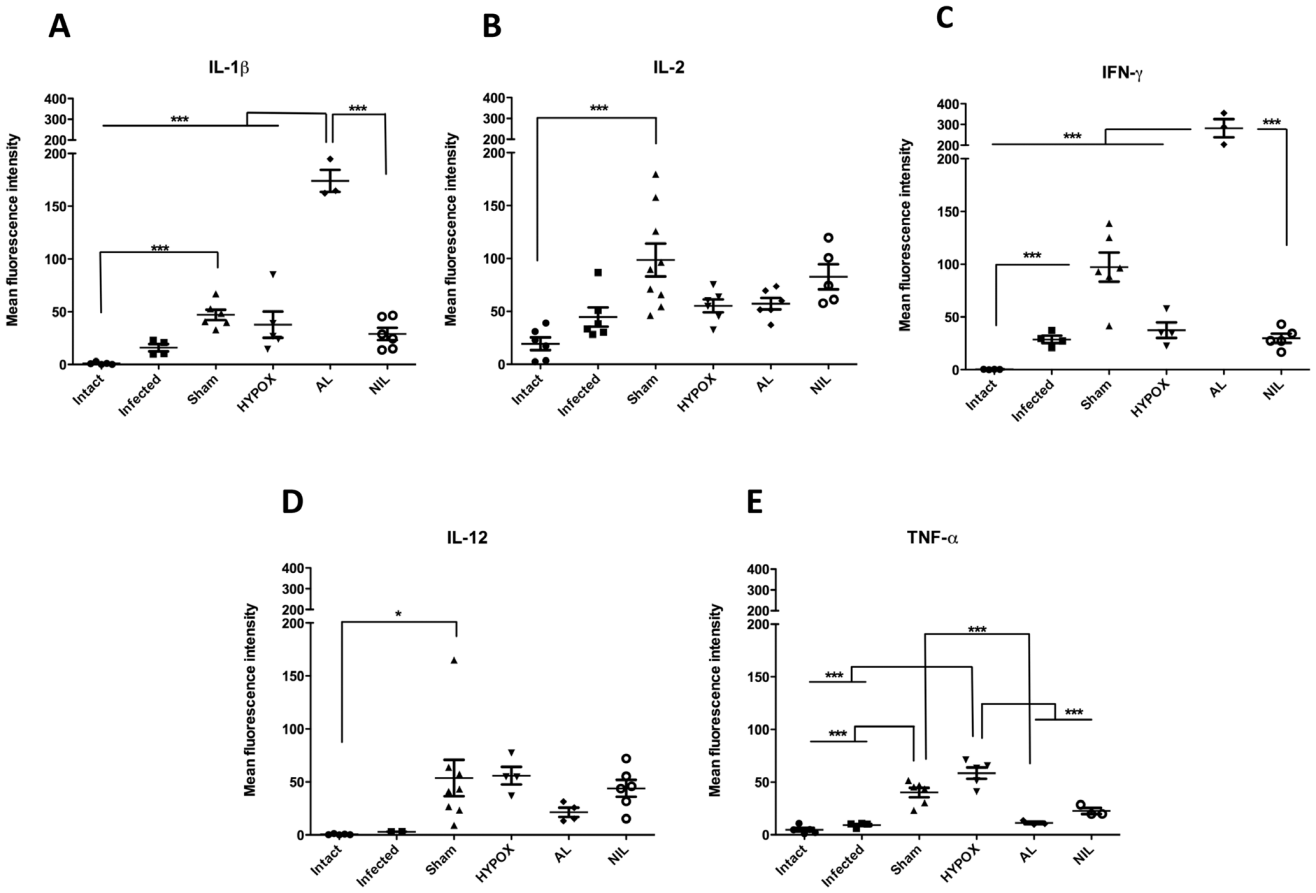
Th1-type related cytokine expression in the duodenum of *T. spiralis* infected hamsters at 5 days pi. Cytokine production was determined by immunocytochemistry and quantitation of its expression was measured as the fluorescence intensity of various cytokines in the small intestine of hamsters subjected to different treatments. Fluorescence intensity data are presented as mean +/− standard deviation. One-way ANOVA. N = 6−9. *** = P<0.001, * = P<0.05.

**Figure 5:**
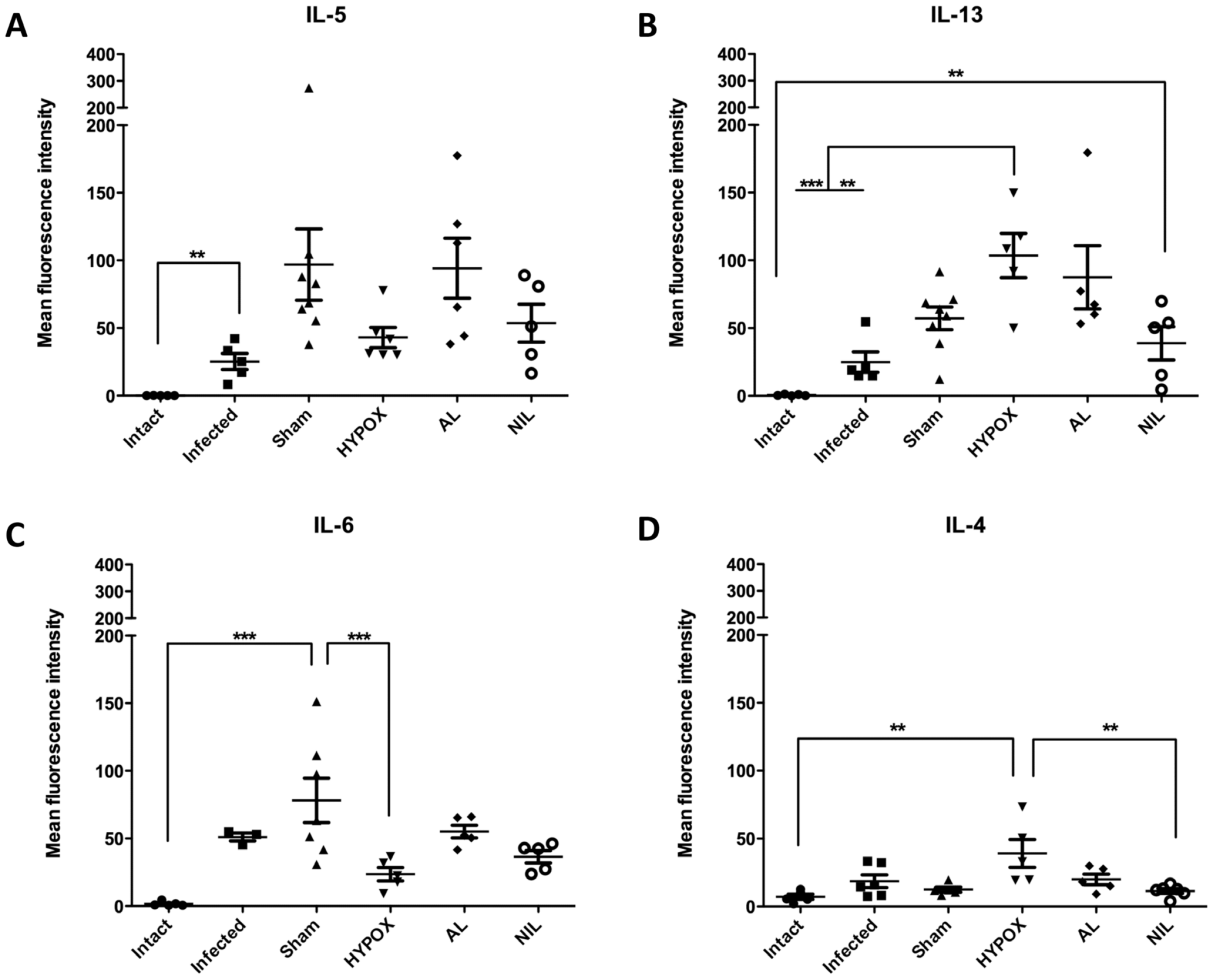
Th2-type related cytokine expression in the duodenum of hamsters experimentally infected with *T. spiralis* at 5 days p. Cytokine production was determined by immunocytochemistry and quantitation of its expression was measured as the fluorescence intensity of various cytokines in the small intestine of hamsters subjected to a variety of treatments. Fluorescence intensity data are presented as mean +/− standard deviation. One-way ANOVA. N = 5−8. *** = P<0.001, ** = P<0.05.

**Figure 6:**
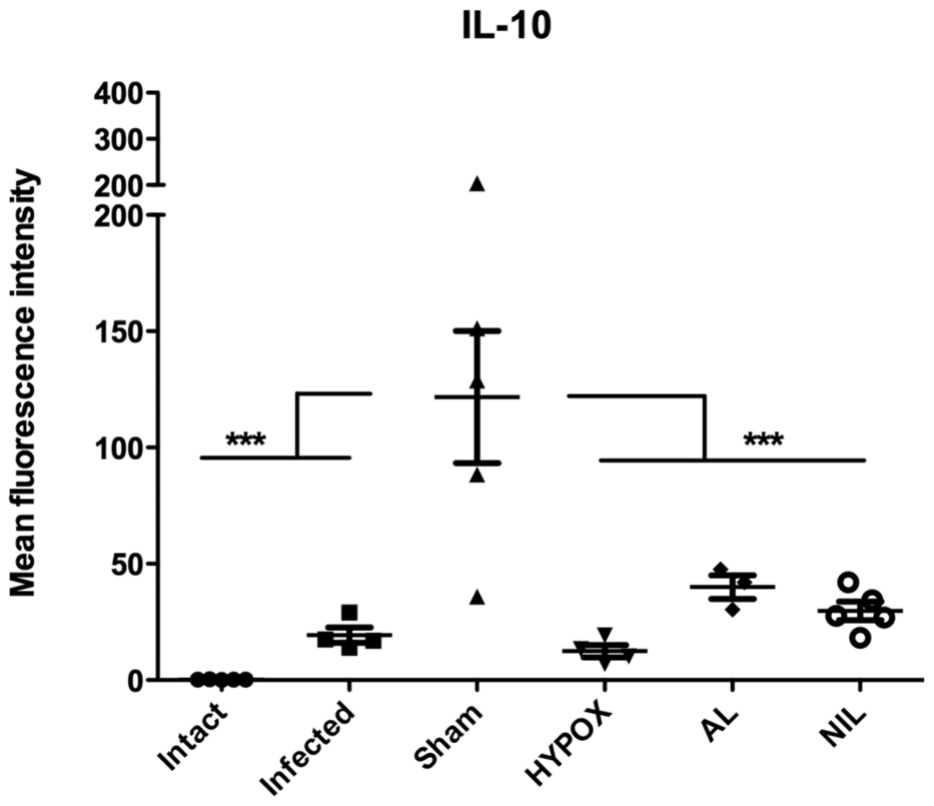
T-reg cytokine (IL-10) expression in the duodenum of experimentally infected hamster with *T. spiralis* at 5 days pi. Cytokine production was determined by immunocytochemistry and quantitation of its expression was measured as the fluorescence intensity of various cytokines in the small intestine of hamsters subjected to a variety of treatments. Fluorescence intensity data are presented as mean +/− standard deviation. One-way ANOVA. N = 4−5. *** = P<0.001, * = P<0.05.

In order to obtain information on the interactions between the intestinal mucosa-gut associated lymphoid tissue and hypothalamic-pituitary axes in animals with different hormonal milieus, the intestinal immunofluorescent expression of the Th1 (IL-1β, IL-2, IL-12, INFγ, and TNFα), Th2 (IL-5, IL-13, IL-4, IL-6), and the T-regulatory IL-10 cytokines, were analyzed in the intestine by immunohistochemistry techniques and assessed with semiquantitative immunofluorescent laser confocal microscopy. Results of the expression of the pro-inflammatory cytokines are shown in the figures 4A, B, C, D and E. Observe that, except for TNFα in the HYPOX group (p<0.001) (Fig. 4E), the comparison between the Intact, Infected, HYPOX, and NIL groups indicate that intestinal fluorescence was poorly developed for the IL-1β, IL-2, INFγ, and IL-12 cytokines. Note in the same figures that, whereas the IL-2, IL-12, and TNFα expressions were also poorly developed in AL animals, a strong and significant fluorescence occurred for the IL-1β and INFγ cytokines. In the SHAM group, an interesting finding occurred; all pro-inflammatory cytokines were mildly but significantly expressed (Figs. 4A, B, C, D and E). For the Th2 cytokines, except for IL-5 and IL-6 in the SHAM group, IL-13 in the HYPOX and AL groups, and IL-4 in the HYPOX group, in which fluorescence was mildly but significantly expressed, fluorescence was poorly developed (Figs. 5A, B, C and D). Figure 6 shows that, except for the SHAM animals, whose fluorescence was significantly increased, IL-10 was poorly expressed.

### Differential Cytokine Immunofluorescence in Lamina Propria and Epithelial Mucosa Layer

Histology of the intestinal mucosa fluorescence also showed a relative but marked difference in cytokines stain intensity between the lamina propria and mucosal epithelial cells of the several experimental groups, suggesting a pituitary-dependent differential distribution of the several cytokines. In comparison with the infected group, the lamina propria of the HIPOX animals showed an increased fluorescence for IL-1β, IL-12, INFγ and TNFα cytokines, whereas a poor or undetected fluorescence was observed in the epithelial mucosa layer. In comparison with the Infected group, AL animals developed a very high accumulation of IL-1β and INFγ in both lamina propria and mucosal epithelium, whereas TNFα was just mildly expressed in the mucosal epithelium. No differences in IL-6 and IL-10 fluorescence were apparent in lamina propria and mucosa epithelial layer of the Infected and AL groups. Also, in comparison with the infected, HYPOX, and AL groups, an increased expression of the cytokines IL-6 and IL-10 was apparent in the lamina propria, but not in the mucosal epithelium layer, of NIL animals.

### Intestinal Histology

**Figure 7:**
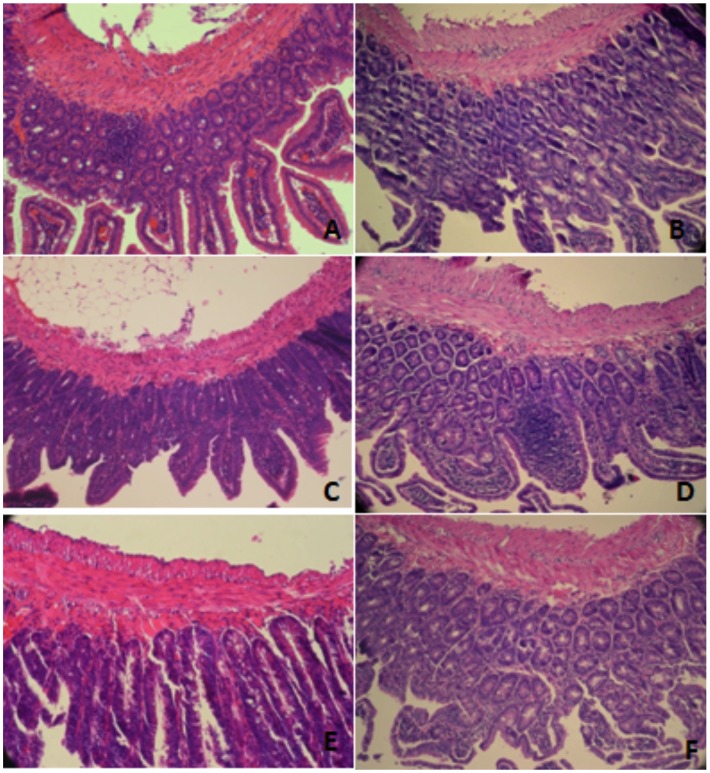
Hematoxylin-eosin stain on histological sections of small intestine of hamsters experimentally infected with *T. spiralis* at 5 days pi. Note the marked hypertrophy microvilli in the Sham group as compared than other groups. (A) Intact; (B) Infected; (C) SHAM; (D) HYPOX; (E) AL; (F) NIL. Arrows in Figures A y C indicate the size increase in intestinal villi, and in D, are to highlight the exacerbated inflammatory infíltrate.

Intestinal histology of the several groups was studied in H-E stained slides. As compared with the Intact and Infected animals, a mild decreased villi height was apparent in HYPOX, AL, and, to a lesser degree, in NIL. In all infected groups, mucosa isolated areas of inflammatory infiltrates were found, indicating the presence a local immune response to the worms (Fig. 7A). Except for the infected animals, in which parasites were found and surrounded by inflammatory cells, no worms were seen in the intestinal mucosa of the remaining infected groups (Figs. 7A and 7B).

## Discussion

The host-parasite relationship implies biochemical co-evolution and cross-talk between both the physiological and metabolic systems [42], [48]. The purpose of this work was to examine the effects of HYPOX, AL, and NIL on the cytokine expression pattern during the immune response against the intestinal infection by *T. spiralis*. Our findings demonstrate that pituitary hormones differentially influence the cytokine expression in spleen and intestine during the intestinal infection by *T. spiralis*. Removal of the pituitary gland (HYPOX) affects the activity of phagocytes, the main cells of the innate immunity involved in killing *T. spiralis* [49], [50]. It has been previously demonstrated that peritoneal macrophages from HYPOX rats show an impaired TNFα response to the *in vitro* stimulation with lipopolysaccharide [30], being also less effective in killing *Salmonella serovar typhimurium* than those cells derived from rats having intact pituitaries [26]. Interestingly, GH injections are able to enhance the resistance of both intact and HYPOX rats following a challenge with *Salmonella serovar typhimurium* [27], [30]. This enhanced resistance correlates with an increased capacity in peritoneal macrophages from those animals to generate reactive oxygen species (ROS), such as superoxide anion and hydrogen peroxide [27]. Additionally, GH is also capable to activate human monocytes for an enhanced intermediate ROS production *in vitro* [51], [52]. We have also reported that HYPOX and NIL, causes increasing bacterial colonization of Peyer patches and spleen, accompanied by a decrease in the intestinal secretory profile of IgA, as well as low IgM and IgG serum levels during the intestinal infection with *Salmonella serovar typhimurium* [1], [3]. All together this information supports the view that both GH and neurohypophysial hormones might play a role as stimulators/modulators of the immune system in peripheral and gut-associated lymphoid tissue (GALT). However, so far there is no information concerning any parasite infection regarding the role of pituitary hormones on both the systemic and intestinal immune regulation. Thus, this is the first set of experiments attempting to demonstrate the role of pituitary hormones in the control of *T. spiralis* or any other helminth.

Decreasing numbers of duodenal adult parasites recovered from our experimental groups (Infected 65%, SHAM 45%, HYPOX 58%, AL 57% and NIL 42%), accompanied by changes in the expression of several cytokines in spleen and intestine, indicate that regardless the change in the hormone and cytokine microenvironment, the innate immune mechanisms remain still effective to eliminate intestinal parasites (Fig. 1).

SHAM animals (with an intact hypothalamic-pituitary axis) exhibited an increase splenic TNFα response, accompanied by a mild but significant increasing in the intestinal expression of Th1, Th2 and T-regulatory (IL-10) cytokines, indicating that a more effective immune response against the intestinal *T. spiralis* infection is induced in SHAM animals. This can be also evidenced by less recovery in the number of intestinal parasites in these animals. It is interesting to note that SHAM animals showed an increase cytokine response against *T. spiralis* than controls, being also possible that such an inflammatory response could be influenced by the mock surgery itself (Figs. 4, 5 and 6).

On the other hand, Infected and HIPOX animals had similar counts of recovered adult worms from the intestine. Interestingly, HIPOX animals exhibited a significant increase in the splenic levels of IL-10, and the intestinal production of TNFα and IL-13, accompanied by a relative enlargement of IL-1β, IL-12, INFγ, and TNFα at the lamina propria. This result indicates that pituitary hormones may not be required for mounting an efficient immune response in HYPOX animals, in order expel the parasite from the host intestine. This result also supports the view that TNFα from spleen and intestinal tissue is important in eliminating this parasite [53], [54].

Interestingly, AL animals show a severe deficiency in anterior pituitary hormones (GH, PRL, ACTH, TSH, FSH, and LH), without alteration in the AVP levels [21]. In comparison with the Infected group, AL animals exhibited similar numbers of recovered intestinal worms (Fig. 1), accompanied by over-expression of IL-1β and INFγ in apparently both the lamina propria and the epithelial mucosa cells (Figs. 4A and 4B). In summary, in AL animals: *i)* no adenohypophysial hormones are present in blood (data not shown), *ii)* circulating AVP is normal, *iii)* no changes in recovered intestinal worms (Control Infected *vs* AL) were apparent, and *iv)* intestinal over-expression of IL-1β and INFγ was developed in response to *T. spiralis* infection. Altogether this information suggests that IL-1β and INFγ might be under the control of neurohypophysial hormones, being not directly involved in the intestinal control of the parasite.

Previously we have reported that NIL rats developed low AVP serum levels [21]. In the present results, significantly less numbers of intestinal adult worms were recovered from NIL hamsters, indicating an increase immune resistance to *T. spiralis* infection. Taking into consideration the splenic increase of TNFα and IL-10 expression levels, as well as the relative intestinal accumulation of IL-10 in the lamina propria, it is possible to speculate that both TNFα and IL-10 could play an important role in parasite elimination, controlling the inflammatory process in the intestine in response to the *T. spiralis* invasion [55]. Since NIL infected hamsters coursed with: *i)* low AVP and oxytocin serum levels, *ii)* functional hypothalamic-adenohypophysial axes, *iii)* increased expression of splenic TNFα and IL-10, and *iv)* increased accumulation of IL-6 and IL-10 in the lamina propria, we are able to propose that these cytokines are under the control of anterior pituitary hormones.

From the present results, we can conclude that: 1) pituitary hormones and hormones from their target glands (adrenal cortex, thyroid gland, and gonads) may not be required for the immune system to generate an adequate immune response in order to expel the parasite, 2) the role of pituitary hormones and hormones from their target glands is to modulate the immune response, and 3) it seems that TNFα and IL-10 are directly involved in the mechanisms of elimination of intestinal parasites.

Most pituitary hormones modulate inflammatory/immune responses, either directly or indirectly. For example, adrenocorticotropin increases the secretion of GCs, which in turn stimulates the immune function at physiological doses [23], [56]–[59]. GH, PRL, TSH, and β-endorphin produced in the anterior pituitary and the AVP released from the posterior pituitary also have immunostimulatory and proinflammatory properties [60]–[62]. Therefore, the differences between NIL and HYPOX hamsters may be related to the amount of hormones located in the anterior and posterior pituitary that regulate the immune response. The fact that AL induced a more marked increase in the inflammatory response against intestinal *T. spiralis* than HYPOX, suggests that the hormones MSH, AVP, and oxytocin from the neurointermediate pituitary lobe may affect both innate and adaptive immune responses. The presence of specific vasopressin receptors and the stimulatory effects of vasopressin on various immune cell subpopulations, including peritoneal macrophages [63], [64], has been described [65]–[72].

Peyer’s patches and the spleen receive catecholaminergic, cholinergic, and peptidergic innervation, as well as an important input from the sympathetic and parasympathetic divisions of the autonomic nervous system [73]. The abundant innervation of the spleen and Peyer’s patches provide the anatomical basis for the interaction between the nervous and immune systems. For example, norepinephrine-containing nerve fibers localized in Peyer’s patches modulate the internalization of pathogenic *Salmonella choleraesuis* into isolated jejunal Peyer’s patches [74]. However, there are no neural pathways directly connecting the hypothalamus with the Peyer’s patches and the spleen. Therefore, the observed effects are probably the result of hormonal influences rather than disruption in neural pathways.

Finally, it is important not to discard a possible direct effect of pituitary hormones on *T. spiralis*, as hormone signaling pathways are conserved and almost ubiquitous in helminth parasites with similar functions to those of invertebrate and vertebrate orthologs. The existence of similar receptors and ligands in the host and the parasite may have major consequences in terms of the establishment and regulation of infections caused by helminths, particularly *T. spiralis*. Indeed, host-derived pituitary hormones may activate parasite receptors and modulate parasite development and differentiation, as already evidenced in most of the parasite models studied, whereas ligand molecules, such as TGF-β may regulate immune responses and inflammation by interacting with host receptors. In this context, the use of parasite molecules mimicking host ligands has already been investigated for the treatment of immunological disorders [75]. Further studies are necessary for a precise characterization of the signaling cascades of these receptors among various parasites, particularly in the case of pituitary hormone-receptor signaling, as only scant information about signaling molecules is available, and mainly refers to flatworm species. It is probably the case and certainly worth investigating, whether *T. spiralis* developed, as a result of many years of co-evolution with its hosts, a complicated host-parasite network clearly affected by hormones, such as pituitary hormones, where all are involved in the maintenance of important parasite processes such as reproduction, differentiation, and infectivity. Likewise, in recent years, much information has been disseminated about how the host hormonal microenvironment may affect many parasite species [42], [76]–[78]. With respect to the significance of the differential effects of hypophysial hormones upon *T. spiralis*, we found it relevant to speculate on the significance of host specificity, when dealing with the parasite’s life cycle. It seems likely that reproductory specialization as described for *T. spiralis* larvae in laboratory hosts could eventually lead to more friendly relations between host and parasite, that is the case among humans, pigs, and *T. spiralis*. Intermediate hosts would thus only permit infection (be selective) on the part of the fittest examples of the parasite (i.e., those inducing lesser immuno-inflammatory responses that might be damaging to both host and parasite) and as a result of their more exploratory behavior, offer parasites more frequently to their definitive predatory host. In the predator’s intestine, the adult stage of the selected parasite would be able to reproduce sexually and diversify into even less irritating versions of the parasite at its larval stage. The larvae would then pass these new improved versions of the parasite onto the proliferation-convenient environment of female mice acting as intermediate hosts. Based on our experiments, it can be concluded that by different mechanisms, hormones from both the anterior and neurointermediate pituitary lobes play an important role in controlling systemic and gastrointestinal immune responses.

However, more experiments are needed to establish the interactions between the hypothalamo-neurohypophysial and immune systems.

The evidence presented in this work illustrates the importance of neuroimmunoendocrine interactions in an immunocompetent host. It strongly suggests an important role for pituitary hormones in the cytokine network involved in parasite elimination. The complexity of the neuroimmunoendocrine interactions suggests that all of the physiological factors (i.e., sex, age, developmental stage) should be taken into consideration for the design of vaccines and new drugs. The pituitary hormonal network appears to offer a possible new therapeutic approach for controlling several immune confrontations, such as trichinellosis by *T. spiralis*, as well as other bowel-related disorders.
